# Supplementary material for: Multiple pathways of toxicity induced by C9orf72 dipeptide repeat aggregates and G4C2 RNA in a cellular model
Source: eLife. 2021 Jun 23;10:e62718. doi: 10.7554/eLife.62718 (PMC8221807; doi:10.7554/eLife.62718)
Supplement: Figure 2—source data 1. [file elife-62718-fig2-data1.docx]

**Numerical values for graph in Figure 2 C**

Percentage of transfected cells with nucleolar NLS-LS. 3 biological repeats, 10 fields of view per repeat, independent transfections per repeat.

|  |  | repeat 1 | repeat 2 | repeat 3 | mean | SD | Number of cells |
| --- | --- | --- | --- | --- | --- | --- | --- |
| Control | -HS | 2.21 | 2.88 | 0.98 | 2.02 | 0.97 | 342 |
|  | +HS | 94.78 | 92.16 | 92.81 | 93.25 | 1.36 | 389 |
|  | +HS +Rec(1h) | 62.96 | 64.29 | 58.23 | 61.83 | 3.19 | 244 |
|  | +HS +Rec(2h) | 21.13 | 15.50 | 16.83 | 17.82 | 2.94 | 372 |
| NES-GA_65_-GFP | -HS | 1.96 | 3.66 | 2.99 | 2.87 | 0.85 | 200 |
|  | +HS | 90.20 | 92.16 | 90.54 | 90.96 | 1.05 | 176 |
|  | +HS +Rec(1h) | 55.88 | 59.46 | 64.15 | 59.83 | 4.15 | 161 |
|  | +HS +Rec(2h) | 27.27 | 21.74 | 29.89 | 26.30 | 4.16 | 233 |
| NLS-GA_65_-GFP | -HS | 2.70 | 1.54 | 2.53 | 2.26 | 0.63 | 218 |
|  | +HS | 96.92 | 92.31 | 98.41 | 95.88 | 3.18 | 284 |
|  | +HS +Rec(1h) | 90.48 | 85.88 | 86.36 | 87.57 | 2.52 | 268 |
|  | +HS +Rec(2h) | 55.95 | 62.90 | 60.00 | 59.62 | 3.49 | 236 |

Two-sided t-test was used to infer significant differences:

+HS +Rec(1h) NLS-GA_65_-GFP vs NES-GA_65_-GFP *p*-Value = 0.0006

+HS +Rec(2h) NLS-GA_65_-GFP vs NES-GA_65_-GFP *p*-Value = 0.0004
