# Supplementary material for: Multiple pathways of toxicity induced by C9orf72 dipeptide repeat aggregates and G4C2 RNA in a cellular model
Source: eLife. 2021 Jun 23;10:e62718. doi: 10.7554/eLife.62718 (PMC8221807; doi:10.7554/eLife.62718)
Supplement: Figure 2—source data 2. [file elife-62718-fig2-data2.docx]

**Numerical values for graph in Figure 2 D**

Viability assay, 6 biological repeats, independent electroporations.

|  | repeat | | | | | |  |  |
| --- | --- | --- | --- | --- | --- | --- | --- | --- |
|  | 1 | 2 | 3 | 4 | 5 | 6 | Mean | SD |
| NES-GA_65_-GFP | 82.42 | 90.78 | 96.05 | 94.84 | 106.86 | 88.91 | 93.31 | 8.22 |
| NLS-GA_65_-GFP | 64.73 | 58.33 | 78.23 | 33.12 | 60.69 | 61.70 | 59.46 | 14.71 |
| NES-β23 | 46.30 | 28.44 | 52.05 | 37.34 | 36.20 | 44.12 | 40.74 | 8.41 |
| NLS-β23 | 105.33 | 116.05 | 93.59 | 66.64 | 81.25 | 96.51 | 93.23 | 17.49 |

Two-sided t-test was used to infer significant differences:

NLS-GA_65_-GFP vs NES-GA_65_-GFP *p*-Value = 0.0006

NLS-β23 vs NES-β23 *p*-Value < 0.0001
