# Supplementary material for: Multiple pathways of toxicity induced by C9orf72 dipeptide repeat aggregates and G4C2 RNA in a cellular model
Source: eLife. 2021 Jun 23;10:e62718. doi: 10.7554/eLife.62718 (PMC8221807; doi:10.7554/eLife.62718)
Supplement: Figure 3—source data 1. [file elife-62718-fig3-data1.docx]

**Numerical values for graph in Figure 3 B**

| -LMB | repeat 1 | repeat 2 | repeat 3 | Mean | SD | Number of cells |
| --- | --- | --- | --- | --- | --- | --- |
| Control | 2.07 | 2.26 | 2.31 | 2.21 | 0.13 | 120 |
| NES-β17 | 1.01 | 0.99 | 1.04 | 1.02 | 0.02 | 70 |
| NES-GA_65_-GFP | 1.34 | 1.16 | 1.28 | 1.26 | 0.09 | 75 |
| NLS-β17 | 1.92 | 2.17 | 2.24 | 2.11 | 0.17 | 80 |
| NLS-GA_65_-GFP | 1.48 | 1.83 | 1.74 | 1.68 | 0.18 | 77 |
| GA_65_-GFP (Cyt) | 1.03 | 1.41 | 1.22 | 1.22 | 0.19 | 72 |
| GA_65_-GFP (Nuc) | 1.79 | 1.78 | 1.49 | 1.69 | 0.17 | 81 |
| GA_65_-GFP-PY | 1.63 | 1.51 | 1.79 | 1.64 | 0.14 | 77 |
|  |  |  |  |  |  |  |
| +LMB | repeat 1 | repeat 2 | repeat 3 | Mean | SD | Number of cells |
| Control | 0.47 | 0.52 | 0.51 | 0.50 | 0.03 | 110 |
| NES-β17 | 0.76 | 0.73 | 0.69 | 0.73 | 0.04 | 75 |
| NES-GA_65_-GFP | 0.61 | 0.72 | 0.61 | 0.65 | 0.06 | 98 |
| NLS-β17 | 0.56 | 0.45 | 0.54 | 0.51 | 0.08 | 79 |
| NLS-GA_65_-GFP | 0.50 | 0.52 | 0.49 | 0.50 | 0.01 | 81 |
| GA_65_-GFP (Cyt) | 0.68 | 0.78 | 0.66 | 0.70 | 0.06 | 93 |
| GA_65_-GFP (Nuc) | 0.53 | 0.47 | 0.55 | 0.52 | 0.04 | 82 |
| GA_65_-GFP-PY | 0.48 | 0.51 | 0.55 | 0.51 | 0.04 | 72 |

Two-sided t-test was used to infer significant differences:

NLS-GA_65_-GFP -LMB vs NES-GA_65_-GFP -LMB p-Value = 0.0225
