# Supplementary material for: Multiple pathways of toxicity induced by C9orf72 dipeptide repeat aggregates and G4C2 RNA in a cellular model
Source: eLife. 2021 Jun 23;10:e62718. doi: 10.7554/eLife.62718 (PMC8221807; doi:10.7554/eLife.62718)
Supplement: Figure 4—source data 1. [file elife-62718-fig4-data1.docx]

**Numerical values for graph in Figure 4 B**

|  | repeat 1 | repeat 2 | repeat 3 | repeat 4 | mean | SD | Number of cells |
| --- | --- | --- | --- | --- | --- | --- | --- |
| Control -TNFα | 2.00 | 1.82 | 2.07 | 1.93 | 1.95 | 0.11 | 518 |
| Control +TNFα | 0.47 | 0.44 | 0.46 | 0.49 | 0.46 | 0.02 | 524 |
| NES-GA_65_-GFP +TNFα | 0.99 | 0.97 | 0.92 | 1.15 | 1.01 | 0.10 | 136 |
| NLS-GA_65_-GFP +TNFα | 0.57 | 0.58 | 0.64 | 0.56 | 0.59 | 0.04 | 101 |
| GA_65_-GFP (Cyt) +TNFα | 1.39 | 1.07 | 0.87 |  | 1.11 | 0.26 | 218 |
| GA_65_-GFP (Nuc) +TNFα | 0.48 | 0.68 | 0.54 |  | 0.57 | 0.10 | 109 |
| GA_65_-GFP-PY +TNFα | 0.52 | 0.54 | 0.50 |  | 0.52 | 0.02 | 127 |
| Htt96Q +TNFα | 0.99 | 1.20 | 1.20 | 0.89 | 1.07 | 0.14 | 120 |

Two-sided t-test was used to infer significant differences:

Control +TNFα vs NES-GA_65_-GFP +TNFα *p*-Value < 0.0001

Control +TNFα vs NLS-GA_65_-GFP +TNFα *p*-Value = 0.0019

Control +TNFα vs GA_65_-GFP (Cyt) +TNFα *p*-Value = 0.0039
