# Supplementary material for: Multiple pathways of toxicity induced by C9orf72 dipeptide repeat aggregates and G4C2 RNA in a cellular model
Source: eLife. 2021 Jun 23;10:e62718. doi: 10.7554/eLife.62718 (PMC8221807; doi:10.7554/eLife.62718)
Supplement: Figure 5—source data 1. [file elife-62718-fig5-data1.docx]

**Numerical values for graph in Figure 5 B**

|  | repeat 1 | repeat 2 | repeat 3 | mean | SD | number of cells |
| --- | --- | --- | --- | --- | --- | --- |
| Control | 0.01 | 0.01 | 0.03 | 0.02 | 0.01 | 479 |
| NES-GA_65_-GFP | 0.12 | 0.05 | 0.11 | 0.09 | 0.04 | 228 |
| NES-G4C2-GFP | 0.55 | 0.52 | 0.68 | 0.58 | 0.08 | 724 |
| NES-β17 | 0.98 | 0.94 | 0.96 | 0.96 | 0.02 | 381 |
| NLS-GA_65_-GFP | 0.09 | 0.21 | 0.24 | 0.18 | 0.08 | 180 |
| NLS-G4C2-GFP | 0.69 | 0.67 | 0.77 | 0.71 | 0.06 | 416 |
| NLS-β17 | 0.11 | 0.14 | 0.07 | 0.11 | 0.03 | 349 |

Two-sided t-test was used to infer significant differences:

NES-GA_65_-GFP vs NES-G_4_C_2_-GFP *p*-Value = 0.0007

NLS-GA_65_-GFP vs NLS-G_4_C_2_-GFP *p*-Value = 0.0007
