# Supplementary material for: Multiple pathways of toxicity induced by C9orf72 dipeptide repeat aggregates and G4C2 RNA in a cellular model
Source: eLife. 2021 Jun 23;10:e62718. doi: 10.7554/eLife.62718 (PMC8221807; doi:10.7554/eLife.62718)
Supplement: Figure 5—source data 2. [file elife-62718-fig5-data2.docx]

**Numerical values for repeats for Figure 5 E**

Data shown in the figure are from repeat 1.

|  | repeat 1 | | repeat 2 | | repeat 3 | |
| --- | --- | --- | --- | --- | --- | --- |
|  | median | number of cells | median | number of cells | median | number of cells |
| Control + CHX | 0.20 | 103 | 0.36 | 65 | 0.31 | 87 |
| Control | 2.38 | 82 | 3.60 | 75 | 3.76 | 62 |
| GFP | 2.36 | 74 | 4.16 | 77 | 3.75 | 61 |
| NES-GA_65_-GFP | 2.74 | 69 | 3.15 | 52 | 2.25 | 21 |
| NES-G_4_C_2_-GFP | 1.45 | 40 | 2.78 | 27 | 2.55 | 36 |
| NLS-GA_65_-GFP | 0.90 | 45 | 1.38 | 51 | 1.38 | 47 |
| NLS-G_4_C_2_-GFP | 0.39 | 46 | 0.81 | 47 | 0.69 | 38 |

Welch’s test was used to infer significant differences:

NES-GA_65_-GFP vs NES-G_4_C_2_-GFP *p*-Value < 0.0001

NES-GA_65_-GFP vs NLS-GA_65_-GFP *p*-Value < 0.0001

NES-G_4_C_2_-GFP vs NLS-GA_65_-GFP *p*-Value = 0.0062

NLS-GA_65_-GFP vs NLS-G_4_C_2_-GFP *p*-Value < 0.0001
