# Supplementary material for: Multiple pathways of toxicity induced by C9orf72 dipeptide repeat aggregates and G4C2 RNA in a cellular model
Source: eLife. 2021 Jun 23;10:e62718. doi: 10.7554/eLife.62718 (PMC8221807; doi:10.7554/eLife.62718)
Supplement: Figure 6—source data 1. [file elife-62718-fig6-data1.docx]

**Numerical values for graph in Figure 6 A**

Viability assay, 5-6 biological repeats, independent electroporations.

|  | repeat 1 | repeat 2 | repeat 3 | repeat 4 | repeat 5 | repeat 6 | mean | SD |
| --- | --- | --- | --- | --- | --- | --- | --- | --- |
| NES-GA_65_-GFP | 82.42 | 90.78 | 96.05 | 94.84 | 106.86 | 88.91 | 93.31 | 8.22 |
| NES-G_4_C_2_-GFP | 52.26 | 50.09 | 35.29 | 49.31 | 41.91 | 31.95 | 43.47 | 8.45 |
| NLS-GA_65_-GFP | 64.73 | 58.33 | 78.23 | 33.12 | 60.69 | 61.70 | 59.46 | 14.71 |
| NLS-G_4_C_2_-GFP | 28.77 | 42.23 | 44.91 | 46.72 | 37.22 | 32.36 | 38.70 | 7.16 |
| GA_65_-GFP | 104.73 | 80.84 | 114.12 | 87.75 | 88.29 | 71.09 | 91.13 | 15.75 |
| G_4_C_2_-GFP | 43.33 | 45.80 | 45.60 | 28.90 | 20.07 | 50.14 | 38.97 | 11.77 |
| PR_73_-GFP | 91.05 | 112.16 | 94.70 | 97.39 | 109.77 |  | 99.48 | 9.22 |
| GR_73_-GFP | 96.33 | 113.15 | 100.70 | 87.10 | 97.13 |  | 104.28 | 7.12 |

Two-sided t-test was used to infer significant differences:

NES-GA_65_-GFP vs NES-G_4_C_2_-GFP *p*-Value < 0.0001

NLS-GA_65_-GFP vs NLS-G_4_C_2_-GFP *p*-Value = 0.0111

GA_65_-GFP vs G_4_C_2_-GFP *p*-Value < 0.0001

G_4_C_2_-GFP vs PR_73_-GFP *p*-Value < 0.0001

G_4_C_2_-GFP vs GR_73_-GFP *p*-Value < 0.0001
